# A Common *CDH13* Variant Is Associated with Low Agreeableness and Neural Responses to Working Memory Tasks in ADHD

**DOI:** 10.3390/genes12091356

**Published:** 2021-08-29

**Authors:** Georg C. Ziegler, Ann-Christine Ehlis, Heike Weber, Maria Rosaria Vitale, Johanna E. M. Zöller, Hsing-Ping Ku, Miriam A. Schiele, Laura I. Kürbitz, Marcel Romanos, Paul Pauli, Raffael Kalisch, Peter Zwanzger, Katharina Domschke, Andreas J. Fallgatter, Andreas Reif, Klaus-Peter Lesch

**Affiliations:** 1 Department of Psychiatry, Psychosomatics and Psychotherapy, Center of Mental Health, University of Würzburg, 97080 Würzburg, Germany; weber_h2@ukw.de; 2 Division of Molecular Psychiatry, Center of Mental Health, University of Würzburg, 97080 Würzburg, Germany; vitale_m@ukw.de (M.R.V.); zoeller_j1@ukw.de (J.E.M.Z.); ku_h@ukw.de (H.-P.K.); kplesch@mail.uni-wuerzburg.de (K.-P.L.); 3 Department of Psychiatry and Psychotherapy, Tübingen Center for Mental Health (TüCMH), University of Tübingen, 72076 Tübingen, Germany; ann-christine.ehlis@med.uni-tuebingen.de (A.-C.E.); andreas.fallgatter@med.uni-tuebingen.de (A.J.F.); 4 Laboratory of Psychiatric Neurobiology, Institute of Molecular Medicine, Sechenov First Moscow State Medical University, 119991 Moscow, Russia; 5 Department of Psychiatry and Psychotherapy, Medical Center—University of Freiburg, Faculty of Medicine, University of Freiburg, 79104 Freiburg, Germany; miriam.schiele@uniklinik-freiburg.de (M.A.S.); katharina.domschke@uniklinik-freiburg.de (K.D.); 6 Center of Psychosocial Medicine, Institute for Sex Research, Sexual Medicine and Forensic Psychiatry, University Medical Center Hamburg-Eppendorf, 20246 Hamburg, Germany; l.kuerbitz@uke.de; 7 Department of Child and Adolescent Psychiatry, Psychosomatics and Psychotherapy, University of Würzburg, 97080 Würzburg, Germany; romanos_m@ukw.de; 8 Department of Psychology I, University of Würzburg, 97070 Würzburg, Germany; pauli@psychologie.uni-wuerzburg.de; 9 Neuroimaging Center (NIC), Focus Program Translational Neuroscience (FTN), Johannes Gutenberg University Medical Center, 55131 Mainz, Germany; rkalisch@uni-mainz.de; 10 Department of Systems Neuroscience, University Medical Center Hamburg-Eppendorf, 20246 Hamburg, Germany; 11 kbo-Inn-Salzach-Klinikum, 83512 Wasserburg am Inn, Germany; peter.zwanzger@kbo.de; 12 Department of Psychiatry, Ludwig-Maximilian-University of Munich, 80336 Munich, Germany; 13 Department of Psychiatry, Psychosomatic Medicine and Psychotherapy, University Hospital Frankfurt, 60528 Frankfurt am Main, Germany; andreas.reif@kgu.de; 14 Department of Translational Neuroscience, School for Mental Health and Neuroscience (MHeNS), Maastricht University, 6229 ER Maastricht, The Netherlands

**Keywords:** ADHD, *CDH13*, neurodevelopment, executive functions, working memory, Big Five, agreeableness

## Abstract

The cell—cell signaling gene *CDH13* is associated with a wide spectrum of neuropsychiatric disorders, including attention-deficit/hyperactivity disorder (ADHD), autism, and major depression. *CDH13* regulates axonal outgrowth and synapse formation, substantiating its relevance for neurodevelopmental processes. Several studies support the influence of *CDH13* on personality traits, behavior, and executive functions. However, evidence for functional effects of common gene variation in the *CDH13* gene in humans is sparse. Therefore, we tested for association of a functional intronic *CDH13* SNP rs2199430 with ADHD in a sample of 998 adult patients and 884 healthy controls. The Big Five personality traits were assessed by the NEO-PI-R questionnaire. Assuming that altered neural correlates of working memory and cognitive response inhibition show genotype-dependent alterations, task performance and electroencephalographic event-related potentials were measured by n-back and continuous performance (Go/NoGo) tasks. The rs2199430 genotype was not associated with adult ADHD on the categorical diagnosis level. However, rs2199430 was significantly associated with agreeableness, with minor G allele homozygotes scoring lower than A allele carriers. Whereas task performance was not affected by genotype, a significant heterosis effect limited to the ADHD group was identified for the n-back task. Heterozygotes (AG) exhibited significantly higher N200 amplitudes during both the 1-back and 2-back condition in the central electrode position Cz. Consequently, the common genetic variation of *CDH13* is associated with personality traits and impacts neural processing during working memory tasks. Thus, *CDH13* might contribute to symptomatic core dysfunctions of social and cognitive impairment in ADHD.

## 1. Introduction

The gene coding for Cadherin 13 (*CDH13*) was found among the top hits in several genome-wide association studies (GWAS) for attention-deficit/hyperactivity disorder (ADHD) [1,2,3]. Additionally, the gene is located within the only significant region identified in a genome-wide linkage meta-analysis [4]. Its recurring association with several other neuropsychiatric disorders, such as autism spectrum disorders (ASD) [5,6], major depression [7,8], bipolar disorder [9], and substance dependence [10,11,12], points to a broad involvement of *CDH13* in neurodevelopmental processes.

*CDH13* SNP variation impacts *CDH13* expression. Drgonova et al. (2016) reported four *CDH13* SNPs in intron 2 within 8 kb, which were associated with *CDH13* mRNA expression levels in the human frontal cortex postmortem tissue. The greatest effect was detected for rs2199430 with an 80% increase in *CDH13* expression levels in samples derived from GG allele carriers [13]. Rs2199430 is located within a 2 kb hotspot of SNPs, which are responsible for the association of *CDH13* with temporal lobe volumes [14], and the minor G allele of rs2199430 was nominally associated (<0.001) with panic disorder in a Japanese cohort [15]. We are following up on these data, which suggest an association of rs2199430 with brain *CDH13* expression levels, brain structure, and brain function by analyzing genotypes on different endophenotypic levels in a large cohort of adult ADHD (aADHD) patients.

*CDH13* is a crucial driver for neurodevelopmental processes [16]. Early studies suggested a negative regulatory role of *CDH13* for axonal guidance in migrating motor neurons by temporal and spatial changes in *CDH13* expression [17]. A recent study supported these findings and pronounced the importance of *CDH13* for the development of serotonergic projections, as *CDH13*^−/−^ mice showed an increased density of serotonergic neurons in the dorsal raphe during brain maturation and adulthood [18]. Phenotypically, *CDH13*^−/−^ mice exhibit locomotor hyperactivity, learning deficits with decreased cognitive flexibility, and memory impairments, hinting at behavioral and executive dysfunctions resembling ADHD symptomatology [19,20]. A remarkable expression pattern with *CDH13* transcripts found in prominent nuclear brain regions, such as the locus coeruleus, the raphe nuclei, and—to a lesser extent—in the ventral tegmental area, underpins the importance of *CDH13* for the modulation of monoaminergic pathways [16,21].

*CDH13* moderates not only rodent but also human behavior. Genetic variation was associated with extremely violent offenses in prisoners [22]. Besides this extraordinary finding at the dark side of human nature, there is evidence for an association of *CDH13* with common personality traits. A *CDH13* SNP was among the best hits for extraversion and agreeableness in a GWAS which investigated the Big Five (openness to experience, consciousness, extraversion, agreeableness, neuroticism) in a population-based sample [23]. As personality disorders and disruptive behavior are frequent in ADHD [24,25], studies of *CDH13* variation in ADHD should consider dimensional personality traits.

*CDH13* is related to executive functioning in childhood ADHD. Arias-Vasquez et al. (2011) showed an association of a SNP in intron 6 with verbal working memory measurements in a case-control sample on a gene-wide level. Furthermore, there was a 72 kb haploblock only 12 kb upstream of rs2199430 with suggestive, though not gene-wide significant, association with verbal memory [26]. This study is the only one that has specifically addressed cognitive endophenotypes in ADHD related to *CDH13* variation in humans so far.

Consequently, there is promising evidence for an association of *CDH13* with neurodevelopmental processes, which might be due to altered neuronal proliferation, synaptic plasticity, and cellular migration during axonal outgrowth. It is unknown whether and how this is reflected by the functional integrity of neural circuitry in humans. Preliminary evidence points to the effect of *CDH13* on personality traits [23] and executive functions [19,26], but no study has searched for an association of *CDH13* with these dimensional endophenotypes in adult ADHD patients. Therefore, we investigated the effect of functional *CDH13* SNP rs2199430 in aADHD on various levels to challenge the hypothesis that common SNP variation in *CDH13* is associated with ADHD beyond the categorical syndrome. First, we performed a case-control association study on the categorical disease level of ADHD. Subsequently, we tested for association with personality traits, as assessed by the NEO-PI-R. Finally, we investigated rs2199430 with regard to task performance in a working memory (n-back) and a continuous performance test (CPT) assessing response inhibition by means of a Go-NoGo task, and measured brain activation as reflected by electroencephalographic (EEG) event-related potentials (ERPs) in response to these tasks in aADHD and control probands.

## 2. Methods

### 2.1. Case-Control Cohort

A sample of 998 adults with an ADHD diagnosis (499 females, 499 males, mean age = 34.4 years, SD = 10.1) was recruited from the in- and outpatient units of the Department of Psychiatry, Psychosomatics, and Psychotherapy, University of Würzburg, Germany. Part of the sample (372 patients recruited between 2003 and 2005) represents the German IMpACT cohort, which has been described previously [27,28]. The extended sample was recruited for a Clinical Research Unit on ADHD (CRU125) [29]. Inclusion criteria were current diagnosis of ADHD according to *DSM-IV* criteria with retrospective confirmation of an onset before the age of 7. Exclusion criteria were IQ below 80, diagnosis of bipolar affective disorder (due to the overlap of clinical symptoms), and better explanation of symptoms by another psychiatric disorder. Control subjects consisted of 884 healthy individuals (498 females, 386 males, mean age = 27.1 years, SD = 7.3) of which 739 volunteers were recruited in the context of the Collaborative Research Center SFB-TRR58 subproject Z02 wave 2 [30,31], and 145 volunteers were recruited within the context of the CRU125. All control individuals were of Caucasian descent and free of current and/or lifetime DSM-IV mental axis I disorders. Written informed consent was obtained from all study participants. The studies were approved by the Ethics Committee of the Universities of Würzburg, Münster, and Hamburg, and all investigations were in accordance with the fifth revision of the Declaration of Helsinki.

### 2.2. Genotyping

DNA was extracted from blood leukocytes by a slightly modified method based on the protocol of Miller et al. [32]. Rs2199430 genotypes were assessed by polymerase chain reaction (PCR) with subsequent restriction enzyme digestion of the PCR product. The digested product was then run on a 4% agarose gel with expected band sizes of 185 bp (AA genotype), 185 + 142 + 43 bp (AG genotype), or 142 + 43 bp (GG genotype). Specificity of the reaction was ensured by sequencing of the PCR product during establishment of the assay. More information about the PCR reaction and primers is given in Supplementary Information (SI) in Supplementary Table S1. Genotypes of the control samples were obtained from Illumina’s PsychChip Array (Illumina, San Diego, CA). Quality control of microarray raw data was performed according to the Ricopili pipeline (https://sites.google.com/a/broadinstitute.org/ricopili/, accessed on 26 August 2021), excluding SNPs with call rate (CR) < 99%, MAF < 1% and SNPs that deviate from HWE (*p* < 1 × 10^−10^) as well as individuals with CR < 95%, outlier from principal component analyses (>3-fold SD from PC1-PC3), gender discrepancies and related or duplicated individuals. To ensure accordance with the genotyping results of our PCR method and the chip array data, 50 samples with available chip data were additionally genotyped by PCR with a 100% match of genotyping results.

### 2.3. Measurement of Personality Traits

Personality traits were measured by means of self-ratings with the NEO-PI-R. The NEO-PI-R questionnaire is a questionnaire that was constructed to reliably assess personality dimensions of the Big Five [33]. For each of the five main categories neuroticism, extraversion, openness to experience, agreeableness, and conscientiousness, 48 items are rated by the probands on a five-point Likert scale (valuated 0, 1, 2, 3, and 4), resulting in a theoretical maximum of raw scores of 192 for each personality dimension. NEO-PI-R data were available from 881 patients and only a small subsample of 134 healthy controls. As the control sample is underpowered to detect the expected small genotype effects on the Big Five traits, we used this sample only for confirmatory analyses in case of positive findings in the larger aADHD sample.

### 2.4. Functional Electrophysiology

N-back and CPT tasks are well-established methods for the investigation of working memory capacity and the execution or inhibition of prepared motor responses as a measure of sustained attention and impulsivity, respectively [34,35]. Both tasks revealed alterations in ADHD vs. control groups in previous studies, with inattentive symptoms negatively influencing n-back performance and high impulsivity disturbing the ability to suppress prepared motor responses, leading to commission errors during CPTs (Go-response on NoGo trials) by hasty acting [36]. Altered event-related potentials (ERPs) during n-back tasks and CPTs measured by EEG have been described as pathophysiological correlates of ADHD both generally [37,38] and in conjunction with copy number and single nucleotide variation of risk genes in ADHD [39,40]. Therefore, we analyzed the association of rs2199430 genotype with task performance and ERPs during an n-back and Go-NoGo (CPT) trial. The EEG sample was a subsample of our case-control cohort from which 193 aADHD patients and 105 healthy controls completed the n-back task and 219 aADHD patients and 109 healthy controls completed the CPT and had viable EEG data (i.e., at least 20 artifact-free epochs per condition). Probands taking any psychotropic medication were excluded from the analysis. ADHD patients were either medication-naive or had to pause psychostimulant treatment at least 3 days prior to testing. As in our previous publications [39,40], the number of omission and commission errors, reaction times to target/Go stimuli, and the standard deviation of reaction times were considered as behavioral data both for the n-back and the CPT. Electrophysiologically, amplitudes of the N200 (at fronto-central electrode positions: F3, Fz, F4, C3, Cz, C4) and P300 (at centro-parietal positions: C3, Cz, C4, P3, Pz, P4) were regarded as indicators of inhibition and (the reallocation of) attention, respectively, for the n-back task. For the CPT, a topographical analysis was conducted focusing on a posterior-anterior shift in the brain electrical field of the P300 from Go to NoGo trials, i.e., the NoGo anteriorization (NGA; see [41]). For more details on the electrophysiological measurements and analyses, please refer to our previous publications [39,40].

### 2.5. Statistical Analysis

Minor allele (G) frequency was 0.333 in the patient and 0.348 in the healthy control group. Rs2199430 genotype distribution did not significantly differ from the Hardy-Weinberg equilibrium, neither in the control (χ^2^-Test, *p* = 0.89) nor in the ADHD group (χ^2^-test, *p* = 0.09). Genotyping call rate was > 99% for both the PCR method and the chip array. χ^2^-tests were performed to analyze associations between rs2199430 genotype and categorical disease states. To account for possible gender effects, χ^2^-tests were carried out separately for female and male participants. As only brain samples from probands homozygous for the minor (G) allele showed increased *CDH13* mRNA levels in a previous study [13], we additionally analyzed a recessive model (GG vs. A allele carriers) with the help of 2 × 2 contingency tables and Fisher’s exact test.

One-way ANCOVAs were conducted to compare the influence of rs2199430 genotype (AA vs. AG vs. GG) on dimensional personality traits as indicated by the Big Five main domains (neuroticism, extraversion, openness to experience, agreeableness, conscientiousness) reflected by respective NEO-PI-R raw scores. After exclusion of only the most extreme values with −3 ≥ Z-scores ≥ +3, test assumptions for the general linear model were met in terms of normally distributed residuals (Shapiro-Wilk test) and homogeneity of variances (Levene’s test). A previous study of the same aADHD cohort showed a strong impact of gender on the NEO-PI-R measures of neuroticism, openness, and agreeableness [29]. Furthermore, there are age- and gender-specific norms for the NEO-PI-R subscales established in various populations [42]. Hence, age and sex were set as covariates.

Statistical analyses of the functional EEG data were performed using IBM SPSS Statistics (version 27). For the n-back task (N200 and P300 amplitudes), repeated-measures ANOVAs were conducted separately for ADHD patients and controls for the 1-back and 2-back task, with anterior-posterior location (N200: frontal vs. central; P300: central vs. parietal row of electrodes), side (left, right, central) and condition (target vs. non-target) as within-subject variables and rs2199430 genotype (AA, AG, GG) as between-subject factor. For the CPT, NGA values—as well as the so-called “centroids” (representing the center of gravity of the P300 brain electrical field in Go and NoGo trials) underlying this difference measure—were compared between genotypes using univariate ANOVAs. Post hoc analyses were conducted using univariate ANOVAs and two-tailed t-tests for paired or independent samples, as appropriate.

The significance threshold was set at 0.05, and all reported *p*-values are nominal, unless stated otherwise.

## 3. Results

### 3.1. Case-Control Association Study

Although there was a nominally lower percentage of GG carriers in the disease group (ADHD 9.9% vs. controls 11.7%, Supplementary Table S2) there was no significant association of rs2199430 genotype with the categorical syndrome level neither in the total sample (χ^2^-Test, *p* = 0.47) nor in the female or male subgroup (*p* = 0.88 and *p* = 0.47, respectively). Also, the recessive model (GG vs. A allele carriers) revealed no significant association with ADHD diagnosis (Fisher’s exact test, *p* = 0.23).

### 3.2. Association of rs2199430 with Personality Traits

**Figure 1:**
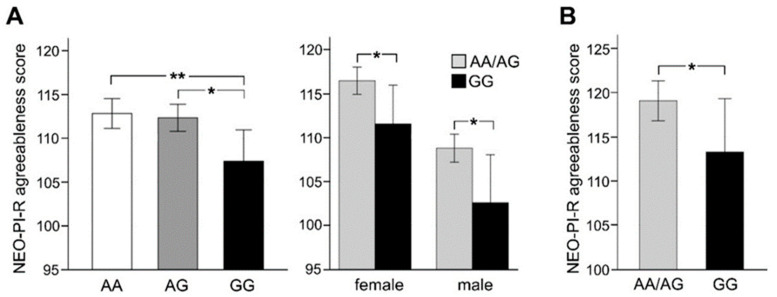
Association of *CDH13* rs2199430 genotype with agreeableness. In the adult ADHD cohort (**A**) *CDH13* rs2199430 genotype was significantly associated with the personality trait of agreeableness as assessed by the NEO-PI-R questionnaire (ANCOVA, *p* = 0.007, 0.035 corr.), which was due to significantly lower agreeableness scores in GG allele carriers as compared to AA and AG allele carriers. Post hoc analyses by t-tests revealed that the effect is present both in the female and in the male subgroup of the aADHD cohort. In the healthy control group (**B**) *CDH13* rs2199430 GG allele carriers likewise exhibited lower agreeableness scores than AA/AG allele carriers. Bars represent means +/− SEM, ** *p* < 0.01, * *p* < 0.05.

ANCOVA with age and gender as covariates showed a significant association of rs2199430 genotype with NEO-PI-R scores for the personality trait of agreeableness (F_2, 871_ = 5.038, *p* = 0.007, η_p_^2^ = 0.011) in the aADHD group. None of the four other personality domains (neuroticism, extraversion, openness to experience, conscientiousness) showed a statistically significant association with rs2199430 genotype (Supplementary Table S3). Bonferroni-adjusted post hoc analyses revealed that GG genotype carriers had significantly lower agreeableness scores (M = 107.4, SD = 16.8) than AG (M = 112.4, SD = 15.7, *p* = 0.016, −5.13, 95%-CI [−9.53, −0.74]) and AA allele carriers (M = 112.8, SD = 16.5, *p* = 0.005, −5.82, 95%-CI [−10.24, −1.39]) as depicted in Figure 1A. One-sided post hoc *t*-tests performed separately in the female and male subgroup revealed that in both females and males the rs2199430 GG carrier group had significantly lower mean agreeableness scores than AA or AG allele carriers (females: t_433_ = 2.10, *p* = 0.019; males: t_441_ = 2.35, *p* = 0.01, Figure 1A). The general difference in agreeableness between females (higher) and males (lower) has already been described elsewhere [29] and therefore was not analyzed here. A 2 × 3 ANOVA showed a weak but significant interaction (F_2, 862_ = 3.69, *p* = 0.024, η_p_^2^ = 0.008) between genotype (GG vs. all A) and ADHD subgroup (inattentive vs. combined vs. hyperactive). Bonferroni-adjusted post hoc *t*-tests revealed that the GG genotype is associated with lower agreeableness in patients with inattentive (t_229_ = 2.9, *p* = 0.004, 0.012 corr.), but not in patients with combined (t_571_ = 2.1, *p* = 0.036, 0.107 corr.), and predominantly hyperactive subtype (t_64_ = −1.1, *p* = 0.275, 0.825 corr.; Table 1). Under the assumption that a general gene-dose effect (higher *CDH13* expression in GG carriers) leads to an association of the rs2199430 GG genotype with lower agreeableness scores in normal populations we performed a one-sided t-test showing a significant association with lower agreeableness scores in GG carriers (*n* = 20, M = 113.3, SD = 13.5) than in AA or AG allele carriers (*n* = 114, M = 119.1, SD = 12.0, t_133_ = 1.95, *p* = 0.027) in the healthy control group (Figure 1B).

### 3.3. Functional Electrophysiology

#### 3.3.1. N-Back (Working Memory)—Behavioral Data

As indicated by univariate ANOVAs carried out separately for ADHD patients and healthy controls, genotype (AA vs. AG vs. GG) did not significantly impact any of the behavioral data (all F < 2.7, *p* > 0.074).

#### 3.3.2. N-Back (Working Memory)—ERP Data

##### N200 Amplitude

**Figure 2:**
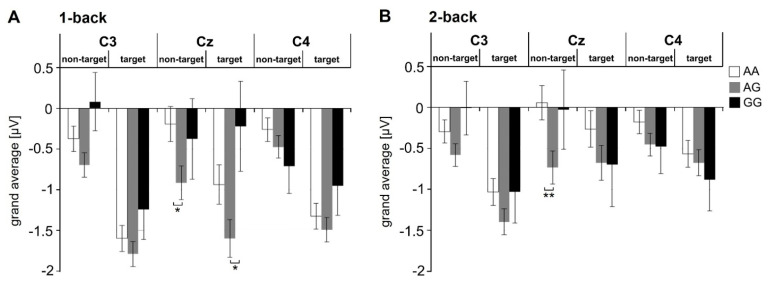
Association of rs2199430 with neural responses to an n-back task. N200 amplitudes associated with *CDH13* rs2199430 genotype (AA, AG, GG) in ADHD patients for target and non-target trials of the 1-back (**A**) and 2-back condition (**B**) at central electrode positions (C3, Cz, C4). A significant heterosis effect of rs2199430 occurred in both conditions with mean amplitudes of AG carriers being significantly higher compared to homozygous patients at central electrode position Cz. Asterisks indicate significant differences between genotypes with ** *p* < 0.01, * *p* < 0.05. Bars represent means +/− SEM.

For ADHD patients, a 3 (genotype: AA vs. AG vs. GG) × 2 (condition: target vs. non-target) × 2 (anterior-posterior position: frontal vs. central electrodes) × 3 (lateralization: left vs. central vs. right electrodes) ANOVA for the n-back data revealed a significant interaction between all four factors for both the 1-back (F_4, 373_ = 3.82, *p* = 0.005, η_p_^2^ = 0.039; Huynh-Feldt corrected due to a violation of the sphericity assumption) and 2-back condition (F_4, 380_ = 2.91, *p* = 0.021, η_p_^2^ = 0.030). Post hoc analyses (univariate ANOVAs) of this interaction showed significant genotype effects only for the more posterior (i.e., central) row, more specifically the middle central electrodes (at Cz for non-target and target trials of the 1-back task as well as for non-target trials of the 2-back task: 2.98 < F_2, 190_ < 3.90, *p* ≤ 0.05, 0.03 < η_p_^2^ < 0.04): N200 amplitudes were consistently higher (i.e., more negative) for the heterozygous genotype (AG carriers) compared to AA (Cz/non-target/1-back: t_175_ = 2.41, *p* = 0.017; Cz/non-target/2-back: t_175_ = 2.71, *p* = 0.007) or GG genotype carriers (Cz/target/1-back: t_106_ = 2.32, *p* = 0.022; Figure 2). In healthy controls, no significant genotype effects were found.

##### P300 Amplitude

**Figure 3:**
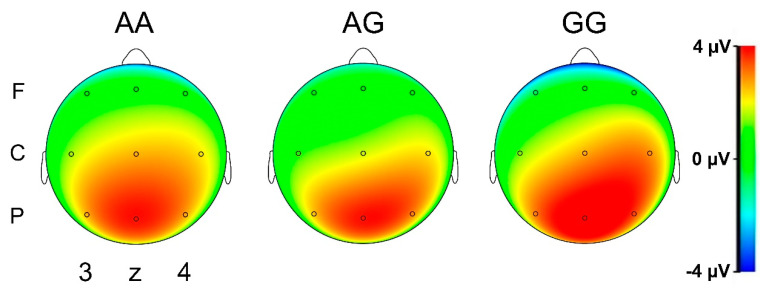
Rightward P300 shift in healthy rs2199430 G allele carriers during CPT target trials. EEG heatmaps for P300 amplitudes in healthy controls with different rs214430 genotypes during target trials of the 2-back condition of the CPT. Maps depict the field distribution of the P300 at the peak of the global field power. G allele carriers exhibit a significant shift of P300 amplitudes to the right compared to individuals with the homozygous AA genotype. F: frontal, C: central, P: parietal, 3: left, z: midline, 4: right.

The ANOVAs for the P300 amplitudes showed no significant genotype effects within the group of patients with ADHD. However, in the healthy control sample, a significant interaction of condition (target vs. non-target) × lateralization (left/central/right electrodes) × genotype (AA/AG/GG) was found for the 2-back condition of the n-back task (F_4, 204_ = 4.12, *p* = 0.003, η_p_^2^ = 0.075). Post hoc analyses of the interaction further revealed no significant genotype effects (univariate ANOVAs) for either target or non-target trials at either left, right or central positions (all F < 1.92, *p* > 0.15); however, depending on the genotype, healthy controls differed in their P300 lateralization pattern: While AA genotype carriers had a clear topographical maximum of P300 amplitudes over central electrode positions (Cz/Pz) as indicated by significant t-comparisons with both left (C3/P3; non-targets: t_48_ = 3.30, *p* = 0.002; targets: t_48_ = 7.22, *p* = 2.35 × 10^−9^) and right sites (C4/P4; non-targets: t_48_ = 3.04, *p* = 0.004; targets: t_48_ = 4.02, *p* = 2.03 × 10^−4^), carriers of at least one G allele did not show this clear central distribution, especially for target trials where amplitudes were equally high for central and right-sided electrode positions, i.e., the topography showed a shift toward the right as indicated by significant t-contrasts of Cz/Pz and C4/P4 as compared to C3/P3: all *t* > 3.2, *p* < 0.01, but no significant differences between Cz/Pz and C4/P4 (Figure 3).

#### 3.3.3. CPT—Behavioral Data

For general comparisons of ADHD patients and healthy controls, see Ziegler et al. (2020). Regarding genetic effects, rs2199430 genotype did not significantly impact any of the behavioral data (number of omission and commission errors, reaction times to Go stimuli, standard deviation of reaction times; control group: all H < 2.2, *p* > 0.30; ADHD group: all H < 2.6, *p* > 0.25).

#### 3.3.4. CPT—Neurophysiological/ERP Data

The NoGo anteriorization (NGA) is a topographical ERP parameter quantifying a frontalization of the brain electrical field of the P300 during inhibitory processes in the context of Go-NoGo tasks [43] that was partly found to be reduced in both adult and childhood ADHD [38,44]. In the present study, the NGA was not significantly affected by rs2199430 genotype (control group: F_2, 106_ = 0.20, *p* = 0.82, η_p_^2^ = 0.004; ADHD: F_2, 216_ = 0.33, *p* = 0.72, η_p_^2^ = 0.003); neither were the Go (control group: F_2, 106_ = 0.27, *p* = 0.77, η_p_^2^ = 0.005; ADHD: F_2, 216_ = 1.64, *p* = 0.20, η_p_^2^ = 0.015) and NoGo centroids (control group: F_2, 106_ = 0.46, *p* = 0.77, η_p_^2^ = 0.014; ADHD: F_2, 216_ = 0.55, *p* = 0.58, η_p_^2^ = 0.005) underlying this difference measure.

## 4. Discussion

*CDH13* intron 2 SNP rs2199430 is associated with frontal cortex *CDH13* expression levels [13]. Here, we show that the functionality of rs2199430 is not restricted to the molecular level but extends to intermediate phenotypes of aADHD. This is underlined by an association of rs2199430 with the personality trait of agreeableness and by allele-specific alterations of neural response patterns during a working memory task in our aADHD cohort. Hence, this study joins the growing line of evidence that the investigation of disease-related intermediate phenotypes is practical for disentangling the contribution of pleiotropic variants to genetically complex neuropsychiatric diseases [26,40,45,46].

Our study revealed no general association of rs2199430 genotype with ADHD on the categorical level. Given the replicated association of *CDH13* with ADHD and the association of rs2199430 with *CDH13* expression levels [1,2,3,4,13], we nevertheless proceeded to test for association of rs2199430 with dimensional personality traits, executive task performance, and electrophysiological parameters.

The association of *CDH13* with agreeableness and extraversion [23], and the frequent comorbidity of ADHD with personality disorders gave reason to investigate the association of rs2199430 with the Big Five personality traits [24,25]. Encouragingly, rs2199430 was associated with agreeableness in the aADHD cohort with GG allele carriers scoring significantly lower than AA or AG allele carriers both in the female and the male subgroup. This genotype effect is underlined by an equally directed effect in a small subsample of healthy controls. When compared to a representative German control population, AA and AG allele carriers from our aADHD cohort are still within the average range in the 38th percentile, whereas GG allele carriers exhibit low mean agreeableness scores ranging in the 24th percentile [47]. ADHD patients generally exhibit lower agreeableness scores than healthy controls, a finding which is pronounced in the hyperactive subtype [29,48]. Therefore, it is not unexpected that the association of the GG genotype with lower agreeableness scores does not appear in the predominantly hyperactive and combined subgroups of ADHD patients in which mean agreeableness scores are significantly lower than those in the inattentive group in general [29].

Low agreeableness has been replicably associated with higher aggression [49,50] and, thus, might build a bridge to disruptive externalizing behavior with detrimental consequences for social and professional relationships common to ADHD [50]. The remarkable association of extremely violent behavior with *CDH13* [22] fits our finding of lower agreeableness in GG carriers, as low agreeableness is an even stronger predictor of violent behavior than the Dark Triad dimensions of Machiavellianism and psychopathy [51]. As higher *CDH13* expression goes hand in hand with lower prefrontal serotonergic innervation [18], the finding of decreased agreeableness in individuals with the high-expression rs2199430 GG genotype is in line with the hypothesis that prefrontal serotonin hypofunction evokes impulsive aggression [52,53]. The other way round, high agreeableness is associated with increased pro-social behavior due to its behavioral facets, such as trust, tender-mindedness, and altruism [54,55]. Thus, it can be speculated that rs2199430 confers a gene dosage-dependent effect of *CDH13* on social behavior. The GG genotype, which is associated with higher frontal cortex *CDH13* expression levels [13] and lower agreeableness, would then hypothetically lead to disturbed or inconsiderate social behavior. This theory matches the observation that conditional *CDH13*^−/−^ mice with Golgi cell-specific knockout show increased reciprocal social interactions [56]. Low agreeableness is commonly found in ASD patients [57,58], and every second child with ASD suffers additionally from ADHD symptoms [59]. Thus, the association of ASD with small copy number variants within the *CDH13* gene [5] warrants further investigation of *CDH13*-dependent alterations in social behavior. Future studies that address the functional effects of *CDH13* in ADHD should consider paradigms of social reciprocity and empathy, for example, by functional imaging studies to test for an association of *CDH13* with pro- and anti-social behavior at a neural system level.

*CDH13* influences working memory performance in childhood ADHD [26]. In contrast to this previous study, we focused on one functional *CDH13* SNP in aADHD. To this end, we assessed working memory performance and EEG neural response patterns during an n-back paradigm. While rs2199430 genotype was not associated with task performance as measured by the number of omission/commission errors and reaction time, we found a significant impact on N200 amplitudes in central electrode positions for both the 1-back and the 2-back condition. This effect only occurred in the aADHD group, but not in the group of healthy controls, possibly indicating disease specificity. Heterozygotes (AG) exhibited significantly higher N200 amplitudes than homozygotes in the central electrode positions. Even if unexpected at first sight, there is a vast amount of literature describing heterosis effects for candidate genes of complex polygenic neuropsychiatric disorders, e.g., for the *BDNF* Val66Met polymorphism [60,61,62], the dopamine transporter, and several dopamine and serotonin receptor genes [63]. Our data can be interpreted as a hint at subtly altered neural processing by a heterosis effect of *CDH13*. The N200 amplitude is generally associated with conflict processing and cognitive inhibition during mentally demanding tasks [64,65]. Hence, it can be argued—in the absence of any behavioral differences between genotype groups—that rs2199430 heterozygotes must invest stronger mental effort during target and non-target trials of both the 1-back and 2-back conditions.

We did not find any genotype-dependent quantitative alterations of P300 amplitudes. However, there was a notable rightward lateralization of P300 amplitudes in rs2199430 G allele carriers, which occurred only in the healthy control but not in the aADHD group. The P300 is thought to reflect stimulus recognition, memory updating, and decision-making during n-back tasks on a higher hierarchical level than the N200, which seems to be more closely related to immediate decision-making [66,67]. Regarding the right lateralization of P300 amplitudes in carriers of at least one G allele (in contrast to no apparent lateralization in the group of AA carriers) in our control group, this effect points toward genotype-based differences in neural processes underlying working memory strategies during the letter n-back task. Specifically, image-based rehearsal strategies have been proposed to rely on the right hemisphere, while “analytic representations” seem to be maintained preferentially in the left frontal areas [68]. Consequently, our data indicate a modifying role of *CDH13* SNP variation in neural processing involved in verbal memory and attentional control. As a previous study hints at an impact of *CDH13* SNP variation on verbal memory in children with ADHD [26], we are deducing that *CDH13* is an important neural moderator for working memory and sustained attention in ADHD. This is in line with the pathophysiological concept of ADHD as a dysexecutive neurodevelopmental disorder with alterations in attentional brain networks with a focus on the connectivity between frontal brain areas, such as the dorsolateral prefrontal cortex and striatal circuits [69].

Behavioral measures and neural responses were not altered by rs2199430 genotype in the CPT (Go-NoGo task). As the CPT mainly assesses prefrontal functions of cognitive response control that are disturbed in highly impulsive individuals, our result is in accordance with reports that *CDH13*^−/−^ mice are predominantly impaired regarding working memory and learning while displaying only subtly increased impulsivity [19,70]. Therefore, future studies that focus on *CDH13* in neurodevelopmental disorders should concentrate on the role of *CDH13*-mediated neuronal outgrowth in attentional networks, such as frontostriatal circuits and hippocampal regions, in both rodent models and human studies. For the former, a conditional knockout mouse is already under investigation [70]; to address the latter, *CDH13*^+/−^ and *CDH13*^−/−^ human-induced pluripotent cell lines have been created recently [71] and can be used to investigate the impact of *CDH13* on the excitatory-inhibitory balance in neuronal cell models [72].

As a limitation, it has to be stressed that the effects of the significant results in this study were only in the small- to medium-sized range and would not survive correction on a gene-wide level, an immanent problem when investigating common SNPs in complex polygenetic neuropsychiatric disorders. Nevertheless, this study provides further evidence that *CDH13*—besides its categorical association with ADHD—influences intermediate phenotypes of dimensional personality traits and executive functions. Thereby, *CDH13* might contribute to important symptomatic domains of cognitive impairment and disturbed social relationships in ADHD. If and how differential brain *CDH13* expression is related to functional alterations in monoaminergic neurotransmission, especially the serotonergic system in this context, should be the focus of future studies.

## Figures and Tables

**Table 1:** ADHD subtype-dependent association of rs2199430 genotype with agreeableness.

	rs2199430 Genotype	*n*	NEO-PI-R Agreeableness	*t*-Statistic	*p*-Value, Corr.
Inattentive Type	AA/AG	209	M = 116.7	t_229_ = 2.9	0.012
			SD = 15.5		
	GG	22	M = 106.7		
			SD = 13.2		
Combined Type	AA/AG	516	M = 111.6	t_571_ = 2.1	0.107
			SD = 16.0		
	GG	57	M = 106.8		
			SD = 18.2		
Hyperactive Type	AA/AG	59	M = 107.5	t_64_ = −1.1	0.825
			SD = 15.9		
	GG	7	M = 114.4		
			SD = 14.7		

The *CDH13* rs2199430 genotype is associated with NEO-PI-R agreeableness scores in adult ADHD patients with the inattentive subtype but not in patients with the combined and predominantly hyperactive/impulsive subtype. *n*: sample size, M = mean, SD: standard deviation, *p*-values are Bonferroni-corrected.

## Data Availability

The datasets used and analyzed for this study are available from the corresponding author upon request.

## Supplementary Materials

The following are available online at https://www.mdpi.com/article/10.3390/genes12091356/s1. Supplementary Table S1: Cycling conditions, primers, and agarose gel image depicting the expected band sizes of the digested PCR product for rs2199430 genotyping; Table S2: rs2199430 genotype distribution in the adult ADHD and control sample; and Table S3: F-statistics for the Big Five personality traits with rs2199430 genotype as an independent variable in the aADHD cohort.
