# Supplementary material for: A Common CDH13 Variant Is Associated with Low Agreeableness and Neural Responses to Working Memory Tasks in ADHD
Source: Genes (Basel). 2021 Aug 29;12(9):1356. doi: 10.3390/genes12091356 (PMC8471784; doi:10.3390/genes12091356)
Supplement: Supplementary file 1 [file genes-12-01356-s001.zip › genes-1342318-supplementary.pdf]

Supplementary Information

A Common *CDH13* Variant is Associated with Low Agreeableness and Neural Responses to Working Memory Tasks in ADHD

Georg C. Ziegler [1,2,*], Ann-Christine Ehlis [3], Heike Weber [1], Maria Rosaria Vitale [2,14], Johanna E. M. Zöller [2], Hsing-Ping Ku [2], Miriam A. Schiele [4], Laura I. Kürbitz [5], Marcel Romanos [6], Paul Pauli [7], Raffael Kalisch [8,9], Peter Zwanzger [10,11], Katharina Domschke [4], Andreas J. Fallgatter [3], Andreas Reif [12] and Klaus-Peter Lesch [2,13,14]

[1] Department of Psychiatry, Psychosomatics and Psychotherapy, Center of Mental Health, University of Würzburg, 97080 Würzburg, Germany; weber_h2@ukw.de
[2] Division of Molecular Psychiatry, Center of Mental Health, University of Würzburg, 97080 Würzburg, Germany; vitale_m@ukw.de (M.R.V.); zoeller_j1@ukw.de (J.E.M.Z.); ku_h@ukw.de (H.-P.K.); kplesch@mail.uni-wuerzburg.de (K.-P.L.)
[3] Department of Psychiatry and Psychotherapy, Tübingen Center for Mental Health (TüCMH), University of Tübingen, 72076 Tübingen, Germany; ann-christine.ehlis@med.uni-tuebingen.de (A.-C.E.); andreas.fallgatter@med.uni-tuebingen.de (A.J.F.)
[4] Department of Psychiatry and Psychotherapy, Medical Center—University of Freiburg, Faculty of Medicine, University of Freiburg, 79104 Freiburg, Germany; miriam.schiele@uniklinik-freiburg.de (M.A.S.); katharina.domschke@uniklinik-freiburg.de (K.D.)
[5] Center of Psychosocial Medicine, Institute for Sex Research, Sexual Medicine and Forensic Psychiatry, University Medical Center Hamburg-Eppendorf, 20246 Hamburg, Germany; l.kuerbitz@uke.de
[6] Department of Child and Adolescent Psychiatry, Psychosomatics and Psychotherapy, University of Würzburg, 97080 Würzburg, Germany; romanos_m@ukw.de
[7] Department of Psychology I, University of Würzburg, 97070 Würzburg, Germany; pauli@psychologie.uni-wuerzburg.de
[8] Neuroimaging Center (NIC), Focus Program Translational Neuroscience (FTN), Johannes Gutenberg University Medical Center, 55131 Mainz, Germany; rkalisch@uni-mainz.de
[9] Department of Systems Neuroscience, University Medical Center Hamburg-Eppendorf, 20246 Hamburg, Germany
[10] kbo-Inn-Salzach-Klinikum, 83512 Wasserburg am Inn, Germany; peter.zwanzger@kbo.de
[11] Department of Psychiatry, Ludwig-Maximilian-University of Munich, 80336 Munich, Germany
[12] Department of Psychiatry, Psychosomatic Medicine and Psychotherapy, University Hospital Frankfurt, 60528 Frankfurt am Main, Germany; andreas.reif@kgu.de
[13] Department of Translational Neuroscience, School for Mental Health and Neuroscience (MHeNS), Maastricht University, 6229 ER Maastricht, The Netherlands
[14] Laboratory of Psychiatric Neurobiology, Institute of Molecular Medicine, Sechenov First Moscow State Medical University, 119991 Moscow, Russia
* Correspondence: ziegler_g@ukw.de; +49-931-201-76387

Cycling conditions

House-made Goldstar 10x Master Mix	2.5 µl
$MgCl_2$ (25mM)	1.0 µl
2,5 mM Nucleotides each	1.0 µl
Primer-hu-Cdh13-F (10µM)	1.0 µl
Primer-hu-Cdh13-R (10µM)	1.0 µl
House-made Taq polymerase	0.3 µl
DNA-Template 1:10	1.0 µl
ddH_20	17.2 µl
Endvolumen	25.0 µl

Rs2199430 primers

Hu-CDH13-rs2199430-F: 5′–AGACATCAGAGGCATTTCCAGG-3′

Hu-CDH13-rs2199430-R: 5′–TCTCCACGTCTTTGTGGTGC-3′

Digest with NEB BbsI-HF #R3539 leads to expected products of the following sizes:

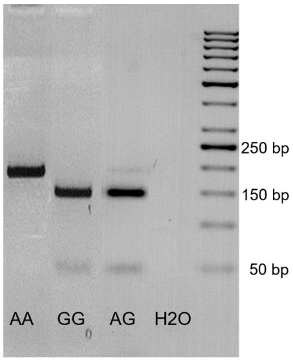

rs2199430 AA 185bp
rs2199430 AG 43bp+142kb+185kb
rs2199430 GG 43bp+142bp

Supplementary Table S1: Cycling conditions, primers, and agarose gel image depicting the expected band sizes of the digested PCR product for rs2199430 genotyping

	AA	AG	GG	sum
**n controls**	481	516	136	884
%	43.0	45.4	11.7	100
**n aADHD**	432	467	99	998
%	43.4	46.8	9.9	100

Supplementary Table S2: rs2199430 genotype distribution in the adult ADHD and control sample. A χ^2-Test showed no significant association of rs2199430 genotype with disease category (aADHD vs. healthy controls, $p = 0.31$).

NEO-PI-R	*rs2199430 genotype*	*n*	*descriptives*	*F-statistics*	*p-value*
neuroticism	AA	378	M = 117.3<br>SD = 25.5	$F_{2,871} = 1.35$	.260
	AG	410	M = 115.1<br>SD = 24.7		
	GG	88	M = 118.8<br>SD = 22.8		
extraversion	AA	378	M = 99.7<br>SD = 22.8	$F_{2,875} = 0.92$	.398
	AG	410	M = 101.5<br>SD = 22.5		
	GG	89	M = 98.9<br>SD = 19.8		
openness	AA	382	M = 111.9<br>SD = 20.7	$F_{2,875} = 0.13$	.879
	AG	409	M = 112.6<br>SD = 19.7		
	GG	89	M = 111.9<br>SD = 17.4		
conscientiousness	AA	381	M = 90.8<br>SD = 23.1	$F_{2,875} = 0.06$	.942
	AG	410	M = 90.6<br>SD = 20.4		
	GG	89	M = 89.7<br>SD = 20.4		
agreeableness	AA	377	M = 112.8<br>SD = 16.5	$F_{2,871} = 5.04$	**.007**
	AG	411	M = 112.3<br>SD = 16.7		
	GG	88	M = 107.4<br>SD = 16.8		

Supplementary Table S3: F-statistics for the *Big Five* personality traits with rs2199430 genotype as independent variable in the aADHD cohort revealing a significant association of

rs2199430 genotype with NEO-PI-R agreeableness scores while the other dimensions remained non-significant.
